# Surface-Deacetylated Chitin Nano-Fiber/Hyaluronic Acid Composites as Potential Antioxidative Compounds for Use in Extended-Release Matrix Tablets

**DOI:** 10.3390/ijms161024707

**Published:** 2015-10-16

**Authors:** Makoto Anraku, Ryo Tabuchi, Shinsuke Ifuku, Takako Ishiguro, Daisuke Iohara, Fumitoshi Hirayama

**Affiliations:** 1 Faculty of Pharmaceutical Sciences, Sojo University, 4-22-1 Ikeda, Nishi-Ku, Kumamoto 860-0082, Japan; E-Mails: anraku@ph.sojo-u.ac.jp (M.A.); sojou_tabuchi@yahoo.co.jp (R.T.); guro2@ph.sojo-u.ac.jp (T.I.); dio@ph.sojo-u.ac.jp (D.I.); 2 DDS Research Institute, Sojo University, 4-22-1 Ikeda, Nishi-Ku, Kumamoto 860-0082, Japan; 3 Graduate School of Engineering, Tottori University, 4-101 Koyama-cho Minami, Tottori 680-8552, Japan; E-Mail: sifuku@chem.tottori-u.ac.jp

**Keywords:** surface-deacetylated chitin nano-fiber, extended-release, interpolymer complex, antioxidant activity

## Abstract

In this study, we examined a possible use of a surface-deacetylated chitin nano-fiber (SDCH-NF) and hyaluronic acid (HA) interpolymer complex (IPC) tablet as a potential antioxidative compound in extended-release matrix tablets. The antioxidant properties of untreated chitin (UCH), SDCH-NF, and HA were examined using *N*-centered radicals derived from 1,1′-diphenyl-2-picrylhydrazyl (DPPH) and 2,2′-azinobis (3-ethylbenzothiazoline-6-sulfonic acid) (ABTS). SDCH-NF and HA had acceptable scavenging abilities and were relatively efficient radical scavengers, but UCH was much less effective. The results suggest that SDCH-NF and HA could serve as scavengers of compounds related to the development of oxidative stress. An SDCH-NF/HA IPC tablet was prepared and evaluated as an extended-release tablet matrix using famotidine (FMT) as a model drug. The release of FMT from the IPC tablet (DCF-NF:HA = 1:1) was slower than that from a SDCH-NF only tablet. Turbidity measurements and X-ray diffraction (XRD) data also indicated that the optimum complexation ratio for IPC between SDCH-NF/HA is 1/1, resulting in a good relationship between turbidity or XRD of the complex and the release ratio of FMT. These results suggest that an SDCH-NF/HA tablet has the potential for use in an extended-release IPC tablet with a high antioxidant activity.

## 1. Introduction

To achieve a constant *in vivo* input rate of freely water-soluble drugs, it is very important to select the most appropriate release-retarding excipients. Hydrophilic polymer gel matrices are widely employed to gain desirable drug release profiles in oral controlled drug delivery fields, because of cost effectiveness and broad regulatory acceptance [1,2]. Interpolymer complexes (IPCs) between polycationic polymers such as chitosan and polyanionic polymers such as alginate are reported to be useful as sustained-release drug matrices than hydrophilic polymer alone [3,4]. Naturally occurring polysaccharides and their derivatives have a bright prospect of constructing IPCs as sustained-release matrices, because of no or minor adverse effects. Among these polysaccharides, chitosan is of interest due to its antioxidant activity. Several studies have shown that chitosan has an ability to scavenge hydroxyl radicals and inhibits the lipid peroxidation of phosphatidylcholine and linoleate liposomes [5,6]. Indeed, we reported in previous papers that chitosan inhibited the peroxidation of human serum albumin (HSA) in both *in vitro* and *in vivo* [7,8,9]. Hyaluronic acid (HA), known as hyaluronan or hyaluronate, is a natural occurring, non-toxic, biocompatible and biodegradable polysaccharide. HA is a unique non-sulfated glycosaminoglycan composed of disaccharide repeating units of β-1,4-d-glucuronic acid and β-1,3-*N*-acetyl-d-glucosamine [10]. Due to the carboxyl groups on the backbone, it is a naturally occurring ionic polysaccharide and has been extensively studied with reference to the formation of nanoparticles with cationic biopolymers, among which chitosan is the most popular one. In fact, HA-based IPC formulations have been proven to be promising biomedical materials, due to their tunable sizes, colloidal stability, low cytotoxicity, protection from enzymatic degradation, *etc.* [11].

Recently, surface-deacetylated chitin nanofibers (SDCH-NF) have received considerable attention, because the cationic amino groups on the fiber surface interact with anionic polymers to form IPCs by electrostatic interactions, endowing the fibers with different physicochemical and biological activities [12,13]. Therefore, they have the potential for use in various fields such as biotechnology, food, cosmetic industries, agriculture, and extreme biomimetics [14,15,16,17]. However, detailed antioxidant activities of SDCH-NF and HA seem to be unavailable so far.

In this study, we examined the possible antioxidant and free radical-scavenging properties of SDCH-NF and HA in *in vitro* studies. Further, we evaluated a combination of SDCH-NF and HA with the antioxidant activity as extended-release matrices.

## 2. Results and Discussion

### 2.1. Scavenging Activity of UCH, SDCH-NF, and HA on DPPH and ABTS Radicals

**Figure 1:**
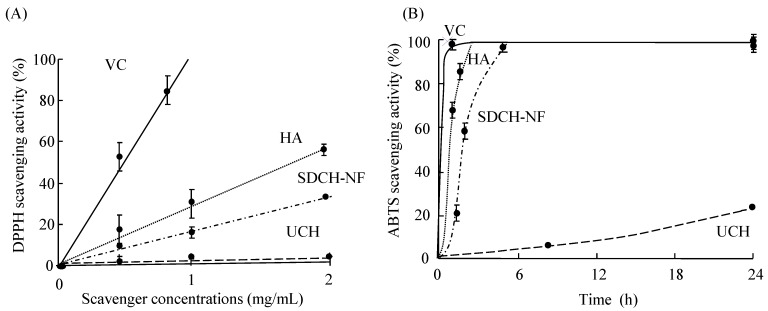
Relative effectiveness of different concentrations of the antioxidants in reducing 1,1′-diphenyl-2-picrylhydrazyl (DPPH) radicals (**A**) and the time course for the reaction of the antioxidants with 2,2′-azinobis(3-ethylbenzothiazoline-6-sulfonic acid) (ABTS) radical cations (**B**). The activities are shown relative to fully reduced DPPH and ABTS (100%). The scavenger concentrations were 5 mg/mL. Ascorbic acid (VC) (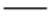), hyaluronic acid (HA) (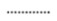) surface-deacetylated chitin nano-fiber (SDCH-NF) (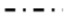), and untreated chitin (UCH) (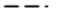).

As shown in Figure 1A, the scavenging activity of UCH, SDCH-NF, and HA on DPPH radicals is substantial and concentration dependent. The antioxidative effect was observed to be in the order of ascorbic acid (VC), a positive control, > HA > SDCH-NF. HA had particularly intense antioxidative effects but did not reach the level of VC. The IC_50_ values for VC, HA, SDCH-NF, and UCH were estimated to be 0.87, 1.69, 3.75, >20 mg/mL, respectively, from the linear plots of Figure 1. The effect of antioxidants on DPPH scavenging is thought to be due to their hydrogen-donating abilities. DPPH is a stable free radical and accepts an electron or a hydrogen radical to form a stable diamagnetic molecule [18]. In the case of ABTS^+^, reduction in the presence of SDCH-NF and HA (2.5 mg/mL) reached 100% within 5 h (Figure 1B). On the other hand, UCH was much less effective. Collectively, these results demonstrate the general ability of HA and SDCH-NF to scavenge oxygen- and nitrogen-centered radicals and suggest that its antioxidant potential, as has been shown in other systems, may be due, at least in part, to this property. Even though the precise mechanism for such radical scavenging activity is not currently clear, it is possible that amino and hydroxyl groups (attached to the C-2, C-3 and C-6 positions of the pyranose ring) react with unstable free radicals, which would facilitate formation of stable macromolecule radicals [19].

### 2.2. Characterization of SDCH-NF/HA Composite

**Figure 2:**
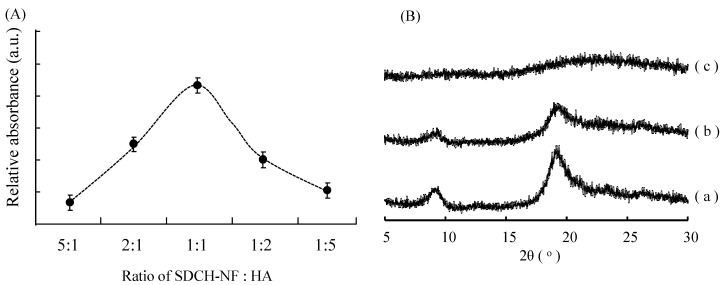
Effect of the ratio of SDCH-NF and HA on the transmittance of the solution (**A**) and powder XRD patterns of SDCH-NF alone (a), SDCH-NF/HA complex (1:1) (b), and HA alone (c) (**B**).

The interaction of SDCH-NF with HA was investigated by monitoring changes in the turbidity of solutions as the result of the precipitation of the IPC. Figure 2A shows changes in the relative absorption at 600 nm as a function of the weight ratio of SDCH-NF/HA. Each SDCH-NF and HA solution was transparent regardless of the concentrations of the components prior to mixing. However, the relative absorption was increased as the result of mixing the SDCH-NF and HA solutions and the absorption reached a maximum at a ratio of 1:1. Further increases in the HA concentration resulted in a decrease in absorbance. These results indicate that SDCH-NF forms a less-soluble IPC with HA predominantly at a 1:1 weight ratio, with micro-particles being precipitated. Since the molecular weights of SDCH-NF and HA employed in this study were approximately 60,000 and 300,000, respectively, the weight ratio of 1:1 corresponded to the 5:1 molar ratio of SDCH/HA, suggesting that one HA molecule interact with five CH molecules. Further, since the molecular weight of *N*-acetylglucosamine is 220 and the deacetylated ratio of SDCH is 20%, the number of the free amino moiety in five SDCH molecules is about 270. On the other hand, HA with a molecular weight of 300,000 contains about 725 units of glucuronic acid units per molecule, based on molecular weights of 194 and 220 for glucuronic acid and *N*-acetylglucosamine, respectively. These results suggest that one anionic carboxylic acid unit in HA molecule interacted with the cationic amino moieties of 2.5–3.0 CH molecules, in agreement with previously reported results for chitosan/sulfobutyl ether β-cyclodextrin (SBE-β-CyD) composites [20].

X-ray diffractomerty (XRD) is a useful tool to study crystal lattice arrangements giving information on various solid characteristics, such as crystallinity, water uptake and the biodegradability of the polymers [21]. The XRD patterns of freeze dried SDCH-NF, HA, and SDCH-NF/HA are shown in Figure 2B. The X-ray diffractogram of SDCH-NF, Figure 2B(a), showed two intensity peaks at 2θ angles of 8.71° and 18.9°. On the contrary, the X-ray diffractogram of HA, Figure 2B(c) shows no sharp reflection counts. The X-ray diffractogram of the SDCH-NF/HA mixture, Figure 2B(b), represents a compilation of the diffraction patterns of SDCH-NF with a measurably lower intensity. The lower intensity of the XRD reached a minimum at a ratio of 1:1 (data not shown). The permanence of these characteristic peaks suggests that the nanofiber structure of SDCH is not greatly disturbed in the SDCH-NF/HA composites.

### 2.3. In Vitro Release Study of Famotidine (FMT) and Scavenging Activity of SDCH-NF/HA/FMT Tablet

**Figure 3:**
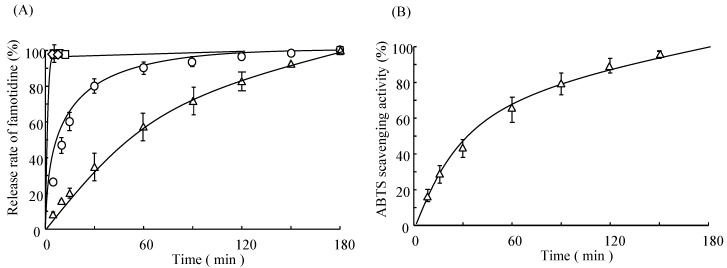
Release profiles of famotidine (FMT) from SDCH-NF/FMT or HA/FMT only, and SDCH-NF/HA/FMT tablet (**A**) and antioxidant activity of SDCH-NF/HA/FMT tablet (**B**) in distilled water. HA only (◊), UCH only (□), SDCH-NF only (○), and SDCH-NF/HA (∆).

Figure 3A shows the release profiles for famotidine (FMT) from matrix tablets containing SDCH-NF/HA/FMT in distilled water. 100% of the drug was released from the UCH or HA only tablet within 0.5 h. On the other hand, in the case of the SDCH-NF only tablet, the rate of drug dissolution was slower. Further, in the case of the SDCH-NF/HA interpolymer complex (IPC) tablet (HA:SDCH-NF = 1:1), the rate of drug dissolution was very slow, compared with that for the HA only, the UCH only or the SDCH-NF only tablets. Further, the markedly slower release reached a maximum at a ratio of 1:1 (data not shown). These results suggest that the SDCH-NF/HA tablet (HA:SDCH-NF = 1:1) could be useful as an extended-release IPC tablet with a high antioxidant activity. In fact, as shown Figure 3B, the SDCH-NF/HA IPC tablet also showed a high scavenging activity for the extended-release of FMT. Given the fact that the SDCH-NF or HA clearly have a high antioxidant activity (Figure 1), the SDCH-NF/HA tablet could be also useful as an extended-release tablet with a high antioxidant activity.

### 2.4. Surface Structure of Tablet before and after Release Study of UCH/FMT, SDCH-NF/FMT, and SDCH-NF/HA/FMT Tablet

**Figure 4:**
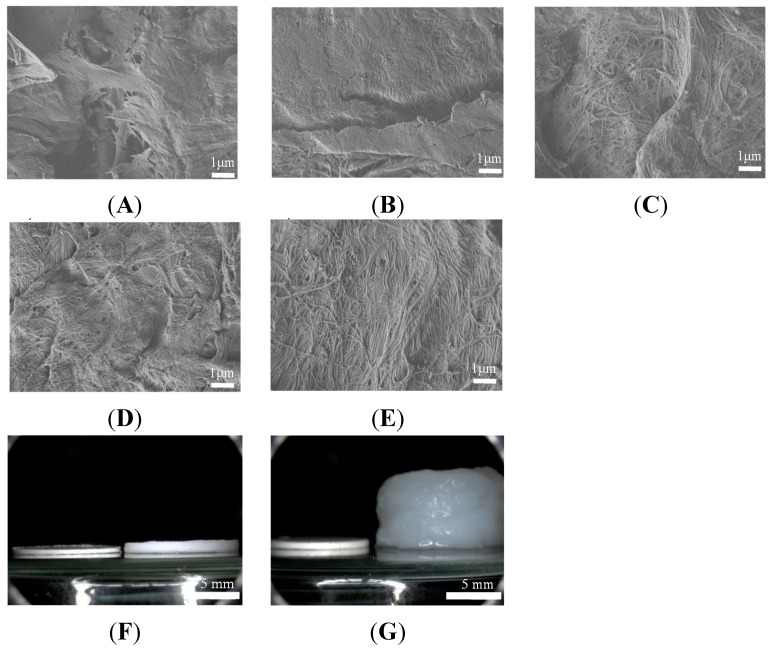
FE-SEM micrographs of surface of CH (**A**), SDCH-NF (**B**,**D**), SDCH-NF/HA tablet (**C**,**E**), before (**A**–**C**) and after (**D**,**E**) release study. Microscopic observations of SDCH-NF only (**left**) and SDCH-NF/HA (**right**) tablets before (**F**) and after (**G**) release study.

Figure 4 shows the morphological changes for the UCH/FMT, SDCH-NF/FMT, and SDCH-NF/HA/FMT tablets before and after their release in water. In Figure 4A, a rough structure can be seen, indicating that the surface of the UCH tablet was roughly crushed before the pre-treatment by the grinder. After one pass of the high-pressure waterjet treatment, the surface of the SDCH-NF tablet was reduced to micro- and nano-sized fibers (Figure 4B). Further, the surface of the SDCH-NF/HA had become fibrillated (Figure 4C). At 3 h after release, the SDCH-NF/FMT was reduced to homogeneous nanofibers (Figure 4D). On the other hand, no significant change in nanofiber structure was found for the SDCH-NF/HA/FMT (Figure 4E). In fact, the SDCH-NF/HA/FMT tablet became more swollen, gelated and expanded about 10 times in thickness from the original size of the tablet, than SDCH-NF/FMT tablet (Figure 4F,G). In addition, no disintegration was observed in the case of the SDCH-NF/HA/FMT tablet, for which a round shape was maintained for the duration of the experiment. In a previous paper, we also reported that SDCH-NF formed strong elastic gels with SBE-β-CyD [20,22]. These results suggest that, after exposure to water, gelation occurred on the surface of the SDCH-NF/HA/FMT tablets, and formed a barrier to famotidine, thus slowing its release. Further, these results indicate that anionic HA markedly reinforced the physical characteristics of the SDCH-NF, owing to the electrostatic interactions between the cationic charges located on the SDCH-NF and anionic charges of the HA molecule which serve to anchor the SDCH-NF into a three dimensional network [22].

Controlled drug delivery systems are to improve various efficacies of drugs, *i.e.*, decrease in undesired side effects and increase in patient compliance [23,24]. Interpolymer complexes (IPCs) have been employed to develop various controlled release tablets, since they have unique properties due to specific electrostatic interaction between oppositely charged polymers, together with other interactions such as hydrogen bonds, van der Waals forces, or hydrophobic interactions [25,26]. For example, poly(acrylic acid) formed IPC with chitosan as the polycationic polymer via electrostatic interactions [27]. Polymer blends combine the attributes of different polymers to give a superior form of dosage [28]. Moreover, polyionic interactions of chitosan with anionic polymers promise to permit the chitosan concentration in a tablet to be considerably reduced [28]. SDCH-NF are considered to have great potential for various applications because they have several useful properties, including a high specific surface area and high porosity [29]. In fact, SDCH-NFs were reported to have numerous bioactivities [30,31,32,33,34,35,36]. For example, SDCH-NFs have been reported to suppress clinical symptoms and colon inflammation in an experimental colitis model [30,31,32]. SDCH-NFs suppress increases in body weight and serum leptin levels in a model of obesity induced by a high-fat diet [28] and decreased serum levels of cholesterol in a rat model of hypercholesterolemia [33]. These results indicate that SDCH-NFs are potentially potent functional foods that can be useful in the treatment of various diseases. Therefore, the formation of SDCH-NF with HA might also permit a more sustained drug release than that using preformed complexes. Thus, a SDCH-NF/HA IPC tablet might be useful as an extended release IPC tablet with a high antioxidant activity.

## 3. Experimental Section

### 3.1. Reagents

Famotidine (FMT) and hyaluronic acid (HA) were purchased from Wako (Tokyo, Japan). Chitin powder (untreated chitin: UCH) from crab shells was purchased from Koyo Chemical. Co., Ltd. (Tokyo, Japan). 1,1′-Diphenyl-2-picrylhydrazyl (DPPH) and 2,2′-azinobis(3-ethylbenzothiazoline-6-sulfonic acid) (ABTS) were supplied by Nacalai Tesque (Kyoto, Japan). All other chemicals were of the highest grade commercially available, and all solutions were prepared using deionized, distilled water.

### 3.2. Preparations of SDCH-NF

The SDCH-NF was prepared using a previously reported procedure with minor modifications [12,13]. UCH (40.0 g) was treated with 20% (*w*/*w*) sodium hydroxide (NaOH, 3.0 L) for 6 h under reflux and an argon atmosphere. After deacetylation, the supernatant was decanted, and the precipitate was thoroughly washed, first with distilled water and then 0.5 wt % aqueous acetic acid by centrifugation to remove the water-soluble products of NaOH, AcONa, and alkaline hydrolyzed chitin. For mechanical disintegration, the deacetylated chitin was dispersed in 4.0 L of aqueous acetic acid and then passed through a grinder (MKCA6-2) at clearance: −1.5 (−1.5 mm), rotating speed: 1500 rpm. The concentration, yield, and degree of deacetylation of the SDCH-NF were 20 wt %. The degree of deacetylation of the SDCH-NF were calculated from both the cationic charges, as determined by an electric conductivity titration method, and nitrogen/carbon contents obtained by elemental analysis using a Thermo Finnigan Flash EA1112 [37].

### 3.3. Preparation of SDCH-NF/HA Tablets Containing Famotidine (FMT)

UCH, SDCH-NF, and HA was dissolved in aqueous acetic acid (1%), and the resulting solutions were freeze-dried. The SDCH-NF/HA mixture solution at a weight ratio of 1:1 was also freeze dried. In the case of SDCH-NF/HA/FMT (0.5:0.5:10 weight ratio), the three components were dissolved in aqueous acetic acid (1%) and freeze dried. The SDCH-NF/HA/FMT composite (22 mg) were compressed, using a hydraulic press with a 10 mm diameter and a 2.0 mm thickness. The compression force was 10 kN/cm^2^ with a dwell time of 1 s.

### 3.4. Scavenging Activity of UCH, SDCH-NF, and HA on DPPH and ABTS Radicals

Radical scavenging activities of different concentrations of polysaccharides were tested in ethanolic solution (10 mL of ethanol, 10 mL of 50 mM 2-(*N*-morpholino) ethanesulfonic acid (MES) buffer (pH 5.5) and 5 mL of 0.5 mM DPPH). Radical scavenging was estimated from the decrease in absorbance of DPPH radicals at 517 nm [38]. Stable ABTS cation radicals were generated by oxidation with potassium persulfate. The reaction mixture contained 200 μL of 70 mM potassium persulfate and 50 mL of 2 mM ABTS in distilled water. Stable ABTS^+^ radicals were generated on standing for 24 h and were used in the assay. The reaction of any antioxidants present with the ABTS^+^ was estimated from the decrease in absorbance at 734 nm [39].

### 3.5. Turbidity Measurements

The turbidity of SDCH-NF/HA suspensions was measured by monitoring the transmittance at a wavelength of 600 nm (Shimdazu UV-1601 spectrophotometer, Kyoto, Japan). Aqueous HA solutions and SDCH-NF in acetic acid (1%) solutions were mixed at different weight ratios, the mixtures were allowed to stand for 10 min, and the transmittance was then measured.

### 3.6. X-ray Diffraction (XRD)

The X-ray diffractrograms of the samples were recorded on an X-ray diffractometer (Rigaku-RINT Ultima+, Tokyo, Japan) under the following conditions: Ni-filtered Cu-Kα radiation (1.542 Å), 40 kV, 40 mA, divergent slit of 1.74 mm (1°), scanning slit of 0.94 mm (1°), receiving slit of 0.15 mm, and goniometer angular increment of 5°/min to determine the physical form of the individual components within the tablet.

### 3.7. Dissolution of Famotidine from Tablets

The rate of dissolution of FMT from tablets was measured using the USP paddle method at 50 rpm using 450 mL of medium at distillated water at 37 °C. An aliquot (1.0 mL) was withdrawn, diluted appropriately with water and analyzed for FMT at a wavelength of 265 nm by means of a UV spectrometer (Shimadzu Scientific Instrument, Kyoto, Japan).

### 3.8. Scanning Electron Microscopy (SEM)

The UCH, SDCH-NF, SDCH-NF/HA suspension was placed in a Teflon petri dish, and ethanol was added. The diluted suspension was dried at 105 °C in the oven, and the obtained sheets were coated with an approximately 2-nm layer of platinum by an ion-sputter coater and observed with a field emission scanning electron microscope (JSM-6700F; JEOL, Ltd., Tokyo, Japan).

### 3.9. Statistics

Statistical significance was evaluated by the 2-tailed paired Student’s *t*-test for comparison between 2 mean values and by ANOVA followed by Newman-Keuls test for comparison among >2 mean values. For all analyses, values of *p* < 0.05 were regarded as being statistically significant. Results are reported as the mean ± SEM.

## 4. Conclusions

Based on the findings presented herein, it can be concluded that SDCH-NF and HA are good scavengers of molecules associated with oxidative stress. The method used to prepare the SDCH-NM/HA/drug composites reported here is simple and convenient, *i.e.*, dissolving three components and freeze drying. Therefore, the findings suggest that the SDCH-NF/HA system could be useful, not only as an extended release IPC tablet with antioxidant activity but also as a safe, non-toxic tablet in general.
